# Phenotypes of adults with congenital heart disease around the globe: a cluster analysis

**DOI:** 10.1186/s12955-021-01696-x

**Published:** 2021-02-10

**Authors:** Edward Callus, Silvana Pagliuca, Sara Boveri, Federico Ambrogi, Koen Luyckx, Adrienne H. Kovacs, Silke Apers, Werner Budts, Junko Enomoto, Maayke A. Sluman, Jou-Kou Wang, Jamie L. Jackson, Paul Khairy, Stephen C. Cook, Shanthi Chidambarathanu, Luis Alday, Katrine Eriksen, Mikael Dellborg, Malin Berghammer, Bengt Johansson, Andrew S. Mackie, Samuel Menahem, Maryanne Caruana, Gruschen Veldtman, Alexandra Soufi, Susan M. Fernandes, Kamila White, Shelby Kutty, Philip Moons, the APPROACH-IS consortium and the International Society for Adult Congenital Heart Disease (ISACHD)

**Affiliations:** 1 Clinical Psychology Service, IRCCS Policlinico San Donato, Milan, Italy; 2 Department of Biomedical Sciences for Health, Università degli Studi di Milano, Milan, Italy; 3 Scientific Directorate, IRCCS Policlinico San Donato, Milan, Italy; 4 Department of Clinical Sciences and Community Health, University of Milan, Milan, Italy; 5 KU Leuven School Psychology and Development in Context, KU Leuven, Leuven, Belgium; 6 UNIBS, University of the Free State, Bloemfontein, South Africa; 7 Peter Munk Cardiac Centre, University Health Network, University of Toronto, Toronto, Canada; 8 Knight Cardiovascular Institute, Oregon Health & Science University, Portland, OR USA; 9 KU Leuven Department of Public Health and Primary Care, KU Leuven, Kapucijnenvoer 35, Box 7001, 3000 Leuven, Belgium; 10 Division of Congenital and Structural Cardiology, University Hospitals Leuven, Leuven, Belgium; 11 KU Leuven Department of Cardiovascular Sciences, KU Leuven, Leuven, Belgium; 12 Department of Education, Toyo University, Tokyo, Japan; 13 Coronel Institute of Occupational Health, Amsterdam UMC, University of Amsterdam, Amsterdam, The Netherlands; 14 Department of Cardiology, Jeroen Bosch Hospital, ‘s Hertogenbosch, The Netherlands; 15 Department of Pediatrics, National Taiwan University Hospital, Taipei, Taiwan; 16 Center for Biobehavioral Health, Nationwide Children’s Hospital, Columbus, OH USA; 17 Adult Congenital Heart Center, Montreal Heart Institute, Université de Montréal, Montreal, Canada; 18 Adult Congenital Heart Disease Center, Helen DeVos Children’s Hospital, Grand Rapids, MI USA; 19 Pediatric Cardiology, Frontier Lifeline Hospital (Dr. K. M. Cherian Heart Foundation), Chennai, India; 20 Division of Cardiology, Hospital de Niños, Córdoba, Argentina; 21 Adult Congenital Heart Disease Center, Oslo University Hospital - Rikshospitalet, Oslo, Norway; 22 Adult Congenital Heart Unit, Sahlgrenska University Hospital/Östra, Gothenburg, Sweden; 23 Institute of Medicine, The Sahlgrenska Academy at University of Gothenburg, Gothenburg, Sweden; 24 Centre for Person-Centred Care (GPCC), University of Gothenburg, Gothenburg, Sweden; 25 Department of Health Sciences, University West, Trollhättan, Sweden; 26 Department of Paediatrics, Queen Silvia Children’s Hospital, Sahlgrenska University Hospital, Gothenburg, Sweden; 27 Department of Public Health and Clinical Medicine, Umeå University, Umeå, Sweden; 28 Division of Cardiology, Stollery Children’s Hospital, University of Alberta, Edmonton, Canada; 29 Monash Heart, Monash Medical Centre, Monash University, Melbourne, Australia; 30 Department of Cardiology, Mater Dei Hospital, Birkirkara Bypass, Malta; 31 Adult Congenital Heart Disease Center, Cincinnati Children’s Hospital Medical Center, Cincinnati, OH USA; 32 Department of Congenital Heart Disease, Louis Pradel Hospital, Hospices civils de Lyon, Lyon, France; 33 Adult Congenital HeartProgram at Stanford, Lucile Packard Children’s Hospital Stanford and Stanford Health Care, Palo Alto, CA USA; 34 Adult Congenital Heart Disease Center, Washington University and Barnes Jewish Heart & Vascular Center, University of Missouri, Saint Louis, MO USA; 35 Adult Congenital Heart Disease Center University of Nebraska Medical Center/ Children’s Hospital and Medical Center, Omaha, NE USA; 36 Taussig Heart Center, Johns Hopkins School of Medicine, Baltimore, USA; 37 Institute of Health and Care Sciences, University of Gothenburg, Gothenburg, Sweden; 38 Department of Paediatrics and Child Health, University of Cape Town, Cape Town, South Africa

**Keywords:** Cluster analysis, Adults with congenital heart disease, Perceived health, Psychological functioning, Health behaviours, Quality of life

## Abstract

**Objective:**

To derive cluster analysis-based groupings for adults with congenital heart disease (ACHD) when it comes to perceived health, psychological functioning, health behaviours and quality of life (QoL).

**Methods:**

This study was part of a larger worldwide multicentre study called APPROACH-IS; a cross sectional study which recruited 4028 patients (2013–2015) from 15 participating countries. A hierarchical cluster analysis was performed using Ward's method in order to group patients with similar psychological characteristics, which were defined by taking into consideration the scores of the following tests: Sense Of Coherence, Health Behavior Scale (physical exercise score), Hospital Anxiety Depression Scale, Illness Perception Questionnaire, Satisfaction with Life Scale and the Visual Analogue Scale scores of the EQ-5D perceived health scale and a linear analogue scale (0–100) measuring QoL.

**Results:**

3768 patients with complete data were divided into 3 clusters. The first and second clusters represented 89.6% of patients in the analysis who reported a good health perception, QoL, psychological functioning and the greatest amount of exercise. Patients in the third cluster reported substantially lower scores in all PROs. This cluster was characterised by a significantly higher proportion of females, a higher average age the lowest education level, more complex forms of congenital heart disease and more medical comorbidities.

**Conclusions:**

This study suggests that certain demographic and clinical characteristics may be linked to less favourable health perception, quality of life, psychological functioning, and health behaviours in ACHD. This information may be used to improve psychosocial screening and the timely provision of psychosocial care.

## Introduction

Congenital heart disease (CHD) is the most common type of birth defect globally. Its prevalence has progressively increased to 9.410/1000 in the period 2010–2017. Since most serious congenital heart defects can be operated on, over 90% of children with CHD now survive into adulthood [1, 2]. The overall prevalence of CHD in the adult population has been estimated to be approximately 3000 per million [3]. In 2014 it had reached 60% of the total CHD population [4]. In those countries where there is improved survival, new challenges must be faced, as the CHD population continues to grow and age. These changing needs encompass, not only to ongoing and lifelong medical issues, for which only palliative rather than curative interventions are available but also difficulties encountered regarding psychosocial well-being [5]. In a recent scientific statement from the American Heart Association on the Diagnosis and Management of Noncardiac Complications in Adults with Congenital Heart Disease, it was observed that these patients are at an increased risk of psychological distress, neurocognitive impairment and social challenges. Furthermore it was recommended that they be screened for psychosocial issues and not only depression [6].

When it comes to perceived health in ACHD (measured with the EQ-5D) [7], there are indications that in this population, the findings are influenced by symptoms, NYHA-classification, age and gender and symptomatic patients reported a lower perceived health on EQ-VAS. In another study from Sweden also using the EQ-5D, worse self-reported health was associated with several medical and social factors; presence of cardiovascular symptoms, active smoking, history of valve surgery, low educational level, and higher systolic blood pressure. [8].

Psychiatric disorders, particularly mood and anxiety disturbances were significantly more frequent in ACHD compared to the general population, and lower cut off scores for the HADS should be utilised for screening purposes [9]. It was also noted that quality of life was independently and negatively associated with a diagnosis of major depression, alcohol dependency, nicotine dependency and NYHA class.

Sense of coherence was reported to be higher in adults with CHD than the general population and was a strong predictor of life quality [10–12]. Independently from patient characteristics, poor illness perceptions (measured by the IPQ-R) were associated with lower quality of life [13].

In a study from Denmark, in which different cardiac populations were compared, including congenital heart disease, predictive factors for worse scores on perceived health status (Short Form-12 and EQ-5D), psychological functioning (HADS), illness perception (Brief Illness Perception Questionnaire) and Qol (HeartQoL) across diagnoses were female sex, older age, being unmarried, planned admission, longer hospital stay, and higher co-morbidity score [14].

A recent extensive review contended that when it comes to quality of life (QoL) measured as life satisfaction (Ex. With the Satisfaction with Life Scale) it is generally good in patients with CHD and can be even better than healthy peers. However, when it is measured as physical functioning, patients with a complex condition do worse than those with a less serious condition or healthy individuals. Predictors of poor QoL were reported to be older age, being a job seeker, unemployed or disabled, never having married, worse functional status, perceived illness and religion and spirituality [15].

Some time ago, a cluster analysis was performed on a smaller sample of ACHD, who were categorised according to their reported good, moderate or poor quality of life, as measured by a linear analogue scale [16]. In this study, most of the patients were found to have a good quality of life (three quarters). Poorer quality of life was associated with a lower educational level, unemployment or disability, associated syndromes, instability of the heart disease, and a poorer functional status.

The aim of the present study was to determine if and what phenotypes could potentially exist in this population. In order to proceed with this, a hierarchical cluster analysis was performed taking into consideration both the primary outcomes (perceived health status, psychological functioning, health behaviours and QOL) and also the secondary outcomes (sense of coherence and illness perception), in order to identify if there are specific associated phenotypes based on sociodemographic and medical variables. A detailed description of primary and secondary outcomes is available in an earlier paper describing rationale, design and method of the study [17].

## Method

An international collaborative research group was created to further study patient reported outcomes in CHD. This study is part of a large study, entitled the Assessment of Patterns of Patient-Reported Outcomes in Adults with Congenital Heart disease-International Study (APPROACH-IS) conducted in partnership with the International Society for Adult Congenital Heart Disease (ISACHD).

APPROACH-IS was a cross sectional study in which data were collected from April 2013 to March 2015 from 15 participating countries over 5 continents: Argentina, Australia, Belgium, Canada, France, India, Italy, Japan, Malta, Norway, Sweden, Switzerland, Taiwan, the Netherlands, and the United States of America (USA) [17].

The study was approved by the institutional review board of the coordinating center (University Hospitals Leuven/KU Leuven, Belgium) and complies with the Declaration of Helsinki. Local institutional board approval was requested and obtained when required. Written informed consent was obtained from all participating patients. More detailed information about the design, rationale and methods of APPROACH-IS is available in a methods paper [17] and the study protocol was recorded at ClinicalTrials.gov: NCT02150603. The current analyses are part of a large project, and the details on the project and publications coming from this project so far can be found using the following link: http://www.approach-is.net/theproject1.html.

This work was supported by the Research Fund—KU Leuven (Leuven, Belgium) through grant OT/11/033 to K.L. and P.M.; by the Swedish Heart–Lung Foundation (Sweden) through grant number 20130607 to M.D.; by the University of Gothenburg Centre for Person-centred Care (Gothenburg, Sweden) to M.D. and P.M.; and by the Cardiac Children's Foundation (Taiwan) through grant CCF2013_02 to H.L.Y. Furthermore, this work was endorsed by and conducted in collaboration with the International Society for Adult Congenital Heart Disease. This study was also partially supported by Ricerca Corrente funding from the Italian Ministry of Health to IRCCS Policlinico San Donato. The authors have no competing interests to declare.

### Study population and procedure

Patients were required to be 18 years or older, diagnosed with CHD and with continuing follow-up and with the capacity to complete self-report questionnaires. More details on patient characteristics and information about the variance with respect to different countries can be obtained from previously published papers [18–20].

#### Measures

The psychometric tests utilised for the primary outcomes can be grouped into four Patient Reported Outcome (PRO) domains (1) perceived health status using the 12-item Short Form Health Survey [21] and the EuroQOL-5D Visual Analog Scale [22]; (2) psychological functioning using the Hospital Anxiety and Depression Scale [23]; (3) amount of exercise per patient (based on the average time in hours, spent per week in various types of physical exercise) using a subscale of Health-Behavior Scale–Congenital Heart Disease [24]; and (4) QoL using a Linear Analog Scale [25]; and the Satisfaction With Life Scale [26].

In addition, two further tests were administered to assess secondary outcomes, the sense of coherence SOC-13 [27] and the Illness Perception Questionnaire Brief IPQ [28]. More details about these measures and the interpretation of the various scores are available in our methodological paper.

For the purposes of the cluster analysis, one measure was selected per construct. For example, when it comes to perceived health, only the EuroQOL-5D Visual Analog Scale was utilised, further supported by EQ-5D validity, reliability and responsiveness previously confirmed in cardiovascular patients and SF-12 in medical populations. Some other dimensions of the SF12, such as the emotional components, are also covered by the inclusion of the other scales.

#### Statistical analysis

A hierarchical cluster analysis was performed by using Ward's method in order to group patients with similar psychological characteristics, which were defined by taking into consideration scores of the following tests: SOC, HBS (physical exercise score), HADS (anxiety and depression subscales), IPQ, SWLS and the Visual Analogue Scale (VAS) scores of the EQ-5D perceived health scale and QoL quality of life.

Stability measure was then compared across clustering methods and numbers of clusters to select the model associated with the most stable solution. Clustering methods considered were the hierarchical Ward’s minimum variance, K-means and d K medians and number of clusters k varying from 2 to 4.

Moreover the number of clusters (k) considered was identified by using the following 3 indices:

1. Cubic clustering criterion (CCC),
2. Pseudo F (PSF),
3. T2 (PST2).

The hierarchical structure of the data was visualized using a dendogram created according to the Ward’s distance. Principal component analysis (PCA) was used to visualize the data for cluster analysis, to characterize the association among psychological tests and to plot together variables and subjects by using a biplot. The biplot was also used to visualize the clusters on two-dimensional plots. The convenience of using two-dimensional plots comes at the expense of the loss of a certain amount of information on the association patterns.

Inferential statistical tests were used to evaluate the association between clusters and variables not used to determine the clusters. In particular, the chi‐square test was used to analyse the association of clusters and categorical variables (demographic and clinical characteristics: gender, education state, cardiac severity and all patient reported outcomes) while the Kruskal–Wallis test was used to analyse the association with continuous parameters (age, number of cardiac surgeries, number of catheterisms). Results were considered statistically significant at *p* < 0.05. Statistical analyses were performed using SAS software, version 9.4 (SAS Institute, Inc., Cary, NC) and with R software version 3.6.3.

## Results

The initial sample consisted in 4028 patients. 260 (6.5%) patients were not included in the data analysis due to missing data. A comparison of demographic and clinical characteristics between those included and those not included in the analysis revealed no evidence of difference between the two groups of subjects (according to Chi-square or Fisher exact test) for all the variables, except for the education level (the percentage of graduates is about 31% for those included and 19% for those not included, while less than High School was 5% for those included and 14% among those not included, *p* < 0.001).

The remaining 3768 patients were divided into three clusters. The PCA analysis showed a positive correlation between the EQ LAS scale and the QoL LAS and between the HADS anxiety and depression subscales. A negative correlation was observed between SOC and HADS anxiety subscale and between IPQ and EQ LAS. A low correlation was shown between SOC and SWLS score.

**Fig. 1:**
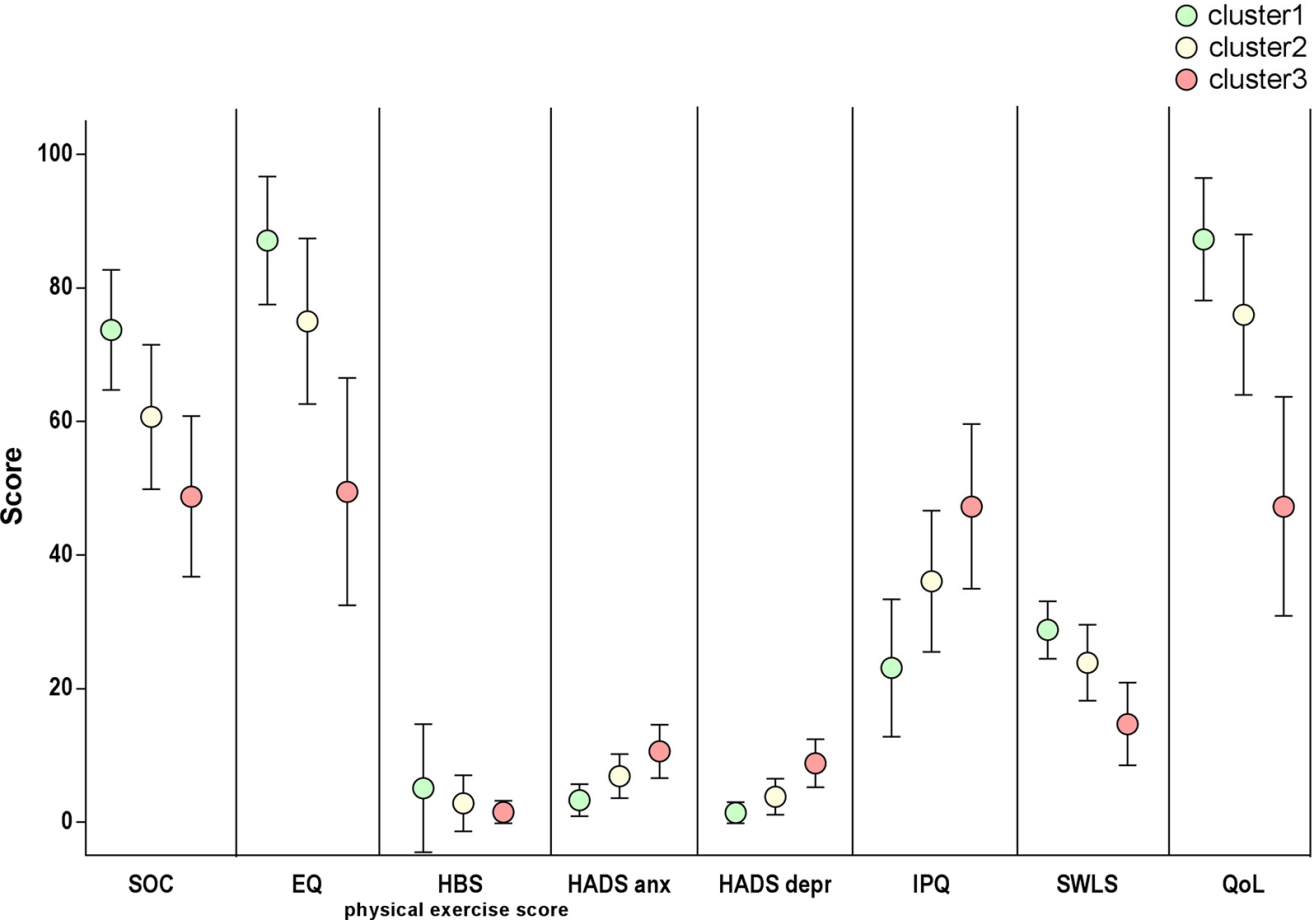
Visual representation of cluster scores

**Fig. 2:**
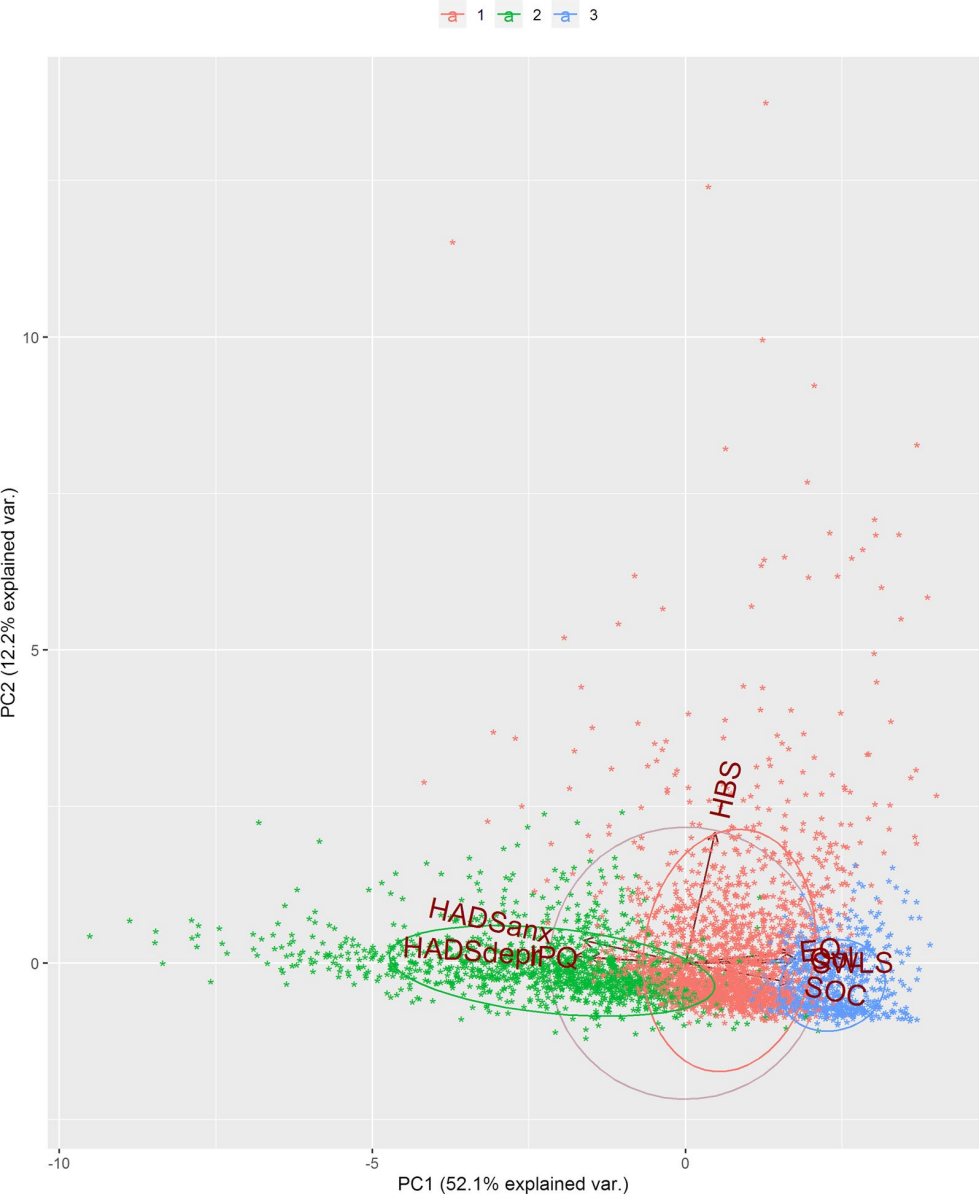
Biplot of the tests of the 3 patient clusters

The distribution of patient scores in the various clusters are reported in Table 1 and a visual representation of these scores can be seen in Fig. 1. Although a trend could be observed regarding physical activity in the various clusters, the HBS (physical exercise score) was not strongly correlated with the results of the other questionnaires (Fig. 2).

**Table 1:** Patients score distribution in clusters

	Cluster (mean ± std–median (25° percentile–75° percentile))		
	1 (N = 1783)	2 (N = 1595)	3 (N = 390)
SOC	73.7 ± 9.0 (75.0 (68.0–80.0))	60.7 ± 10.8 (61.0 (54.0–68.0))	48.8 ± 12.0 (48.0 (42.0–56.0))
EQ	87.1 ± 9.6 (90.0 (80.0–95.0))	75.0 ± 12.4 (75.0 (70.0–82.0))	49.5 ± 17.0 (50.0 (40.0–60.0))
HBS Physical exercise score	5.1 ± 9.6 (1.3 (0–6.2))	2.8 ± 4.2 (0.3 (0–4.5))	1.5 ± 1.7 (0 (0–0.9))
HADS anx	3.3 ± 2.4 (3.0 (1.0–5.0))	6.9 ± 3.3 (7.0 (5.0–9.0))	10.6 ± 4.0 (10.5 (8.0–14.0))
HADS depr	1.4 ± 1.6 (1.0 (0–2.0))	3.8 ± 2.7 (3.0 (2.0–5.8))	8.8 ± 3.6 (9.0 (6.0–11.0))
IPQ	23.1 ± 10.3 (23.0 (16.0–30.0))	36.1 ± 10.6 (37.0 (29.0–44.0))	47.3 ± 12.3 (48.0 (41.0–56.0))
SWLS	28.8 ± 4.3 (29.0 (27.0–32.0))	23.9 ± 5.7 (25.0 (20.0–28.0))	14.7 ± 6.2 (14.0 (10.0–19.0))
QoL	87.3 ± 9.2 (90.0 (80.0–95.0))	76.0 ± 12.0 (78.0 (70.0–85.0))	47.3 ± 16.4 (50.0 (39.0–60.0))

Data are presented as: mean ± standard deviation and (median, interquartile range) or n (%)

*SOC* Sense of Coherence, *EQ* EuroQol Numerical Rating Scale, *HBS* Health Behavior Scale, *HADS* Hospital Anxiety and Depression Scale (*anx *Anxiety, *depr* depression), *IPQ* Illness Perception Questionnaire, *SWLS* Satisfaction with Life Scale, QoL: Quality of Life Numerical Rating Scale

Regardless of stability indices and number of clusters, more stable solutions were obtained with hierarchical Ward’s model. In addition, the most stable solution was obtained with 2 clusters but coefficient of 3 clusters were similar (hierarchical cluster 2: APN = 0.0009, AD = 40.26, ADM = 0.19, FOM = 10.06, Connectivity = 9.32, Dunn = 0.15 and Silhouette = 0.62 while cluster 3: APN = 0.0057, AD = 40.20, ADM = 0.46, FOM = 10.03, Connectivity = 9.32, Dunn = 0.15 and Silhouette = 0.60). Therefore, we decided to compute with a hierarchical algorithm and a predefined number of clusters equal to 3 which included a large number of patients.

**Fig. 3:**
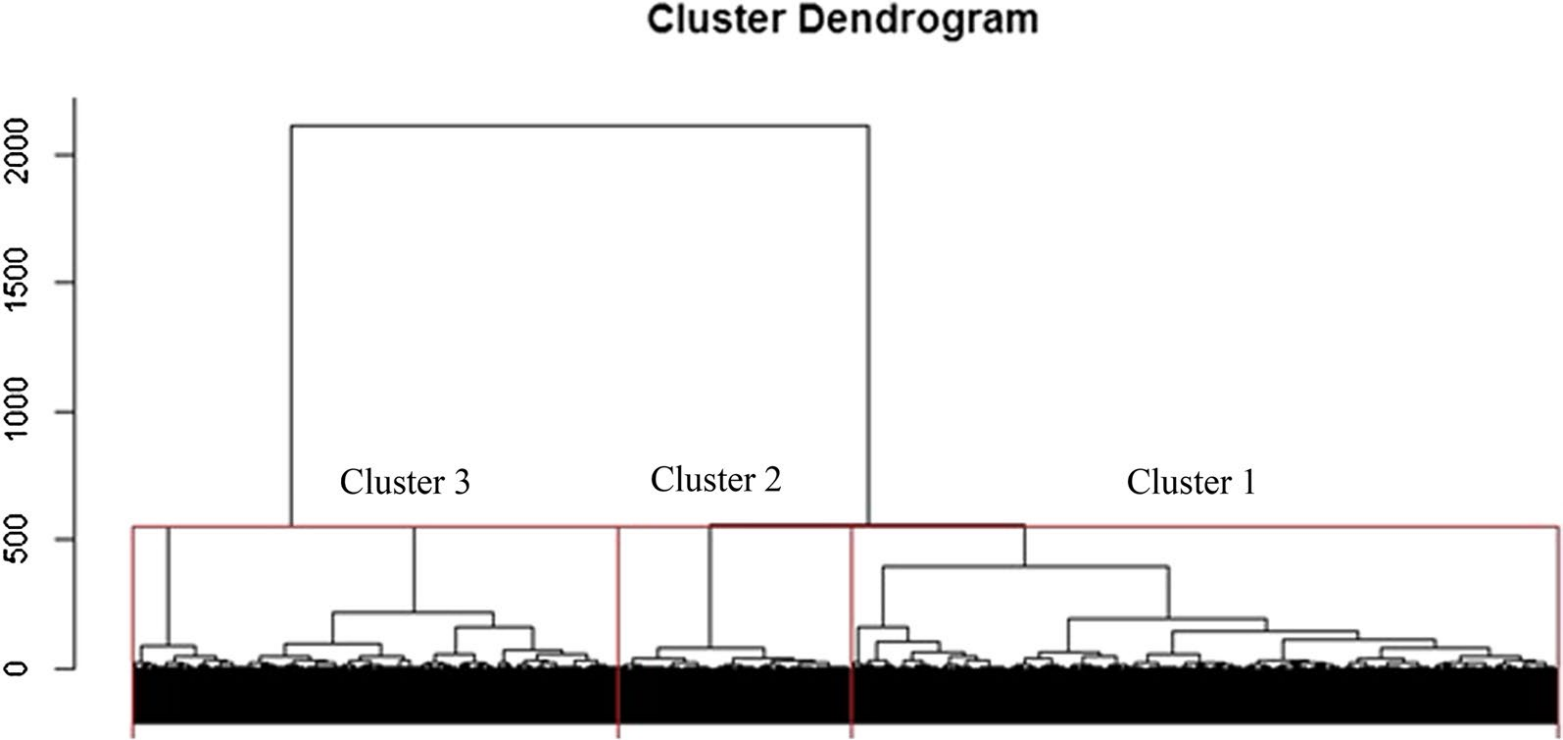
Single linkage clustering

The first and second cluster represented 89.6% of patients in the analysis. Cluster 1, 2 and 3 included 1783, 1595 and 390 patients (Fig. 3) respectively. When it comes to a Sense of Coherence and coping styles (SOC) in the first and second cluster, the patients reported above average scores (73.7 ± 9.0 and 60.7 ± 10.8 respectively) whereas patients in the 3rd cluster reported scores that were generally in the average range (48.8 ± 12.0).

Finally, the Health Behaviour Scale (HBS) (physical exercise score) mean was 5.1 ± 9.6 for cluster 1, 2.8 ± 4.2 for cluster 2 and 1.5 ± 1.7 for cluster 3. This indicates that the patients in the first cluster were more active physically, the ones in the second cluster were reasonably active, but less so than cluster 1, and the patients in the third cluster were the least active physically, when measured by number of hours of sports/physical activity per week. However, we have to remark, that even though the median HBS in the first cluster is higher than the other clusters, there is a large variability in the scores, indicating a degree of overlap with the other clusters.

The prevalence of males decreased through clusters (51.0% in cluster 1 vs 45.0% in cluster 2 vs 42.3% in cluster 3, *p* < 0.0001) (Table 2). Patients in cluster 1 had significantly higher levels of education than patients in cluster 3 (college and university 55.7% vs 53.4% vs 34.5%). The complexity of CHD varied significantly in the 3 clusters as follows: simple lesions in 29.1% vs 24.0% vs 19.7%; complex lesions in 20.8% vs 28.0% vs 35.6% *p* < 0.0001). Current congestive health failure increased significantly from cluster 1–3 (1.4% vs 4.1% vs 9.4% *p* < 0.0001) as well as the prevalence of a cardiac device (8.6% vs 13.4% vs 18.9% *p* < 0.0001). History of arrhythmia increased significantly from cluster 1 to 3 (22.8% vs 29.15 vs 42.4% *p* < 0.0001). The subgroups were statistically associated with the prevalence of admission in hospital for cardiac disease within the past year (13.5% vs 17.7% vs 30.5% *p* < 0.0001), with the number of cardiac surgeries (1.4 ± 1.3 vs 1.7 ± 1.5 vs 1.0 ± 1.6 *p* < 0.0001) and number of catheterizations (0.6 ± 1.2 vs 0.9 ± 1.5 vs 1.0 ± 1.6 *p* < 0.0001). There was a significant difference in the prevalence of reported mood (cluster 1 2.9% vs cluster 2 6.5% vs cluster 3 20.4% *p* < 0.0001) and anxiety disorders (respectively 1.6% vs 6.0% vs 13.6% *p* < 0.0001).

**Table 2:** Demographics and clinical characteristics of the clusters

	Cluster			*p* value
	1	2	3	
Complexity				< 0.0001
Simple	519 (29.1)	382 (24.0)	77 (19.7)	
Moderate	893 (50.1)	761 (47.7)	174 (44.6)	
Great	371 (20.8)	452 (28.3)	139 (35.6)	
Gender				0.0002
Male	905 (51.0)	715 (45.0)	164 (42.3)	
Educ				< 0.0001
Less than high school	64 (3.6)	87 (5.5)	37 (9.5)	
High school	718 (40.7)	651 (41.2)	217 (55.9)	
College degree	380 (21.5)	373 (23.4)	53 (13.7)	
University degree	603 (34.2)	471 (29.8)	81 (20.9)	
Congestive heart failure				< 0.0001
Never	1627 (92.8)	1375 (87.6)	299 (78.1)	
Past, not current	101 (5.8)	131 (8.3)	48 (12.5)	
Current	25 (1.4)	64 (4.1)	36 (9.4)	
Cardiac device				< 0.0001
None	1391 (91.5)	1265 (86.6)	283 (81.1)	
ICD	45 (3.0)	57 (3.9)	18 (5.2)	
PM	85 (5.6)	139 (9.5)	48 (13.8)	
History of arrhythmia	404 (22.9)	462 (29.1)	165 (42.5)	< 0.0001
Cognitive impairment	16 (0.9)	23 (1.5)	4 (1.0)	0.33
Inpatient cardiac adm within past year	236 (13.5)	278 (17.7)	117 (30.5)	< 0.0001
Mood disorder	52 (2.9)	103 (6.5)	79 (20.4)	< 0.0001
Anxiety disorder	29 (1.6)	96 (6.0)	53 (13.6)	< 0.0001
Age*	34.7 ± 12.8 (31.0, 25.0–42.0)	33.7 ± 12.2 (31.0, 24.0–40.0)	36.9 ± 13.1 (34.0, 26.0–46.0)	< 0.0001
Number of cardiac surgeries*	1.4 ± 1.3 (1.0, 1.0–2.0)	1.7 ± 1.5 (1.0, 1.0–2.0)	2.0 ± 1.7 (2.0, 2.0–3.0)	< 0.0001
Number of interventional caths*	0.6 ± 1.2 (0.0, 0.0–1.0)	0.9 ± 1.5 (0.0, 0.0–1.0)	1.0 ± 1.6 (0.0, 0.0–1.0)	< 0.0001

Data are presented as: mean ± standard deviation and (median, interquartile range) or n (%)

Data are presented as: mean ± standard deviation and (median, interquartile range) or n (%).

*SOC* Sense of Coherence, *EQ* EuroQol Numerical Rating Scale, *HBS* Health Behavior Scale, *HADS* Hospital Anxiety and Depression Scale (*anx *Anxiety, *depr* depression), *IPQ* Illness Perception Questionnaire, *SWLS* Satisfaction with Life Scale, QoL: Quality of Life Numerical Rating Scale

## Discussion

The ACHD population is a growing population with specific medical and psychosocial challenges. In order to address these needs, it is extremely important to identify factors associated with poorer outcomes. Although numerous studies have explored perceived health status, psychological functioning, quality of life and health behaviours, different instruments were used, making it difficult to compare results in a large subset of patients. Most studies did not include a sufficiently large population to conduct a cluster analysis, especially one that considers the four domains selected in our study [17].

In the current analysis, identification of 3 clusters permitted inclusion of the majority of patients (93.5%) with significant differences between all 3 of them. The first and second clusters included the majority of the study population with favourable PROs, with the first cluster reporting the best scores. A smaller portion of the study population i.e. 390/3768 (10.3%), had worse PRO in the various domains. In the following paragraphs we will discuss the various domains and compare them with the available studies using the same instruments.

When it comes to perceived health, there have been contradictory results. As noted in the Introduction, perceived health as measured by the EQ-5D was linked with the following; symptoms, NYHA, age and gender, presence of cardiovascular symptoms, active smoking, history of valve surgery, low educational level, and higher systolic blood pressure [7, 8].

Our study is in line with these results. First of all, results are consistent with other studies in which perceived health is linked to quality of life. In the third cluster there was a substantial difference when it comes to the VAS scores in health perception. Consistent with previous studies, there are more females in this cluster, their average age is significantly higher, their education is significantly lower and there were significantly higher prevalence of congestive heart failure and arrhythmia. The 3^rd^ cluster also had the largest percentage of complex heart disease and patients admitted to the hospital in the previous year, with the lowest physical exercise scores of the HBS.

The results in this study are also concordant with a study from Belgium on a smaller sample of ACHD [16]. Similar to our study, most patients were found to have a good quality of life (three quarters) Lower educational level, unemployment or disability, associated syndromes and functional cardiac outcomes were associated with poorer quality of life. The clustering of the patients also support the results on psychological functioning, where the HADS was utilised [9]. In the third cluster with the lowest quality of life scores, there was a much higher prevalence of mood and anxiety disorders, also reported by a significant difference in the HADS scores, where most of the patients reported a mild depression and a mild to moderate anxiety.

As expected, when reviewing the secondary and explanatory variables, sense of coherence and illness perception, there was an important difference in the clusters. Patients in the third cluster had substantially a lower sense of coherence scores than the other 2 clusters. They also reported the highest illness perception scores, indicating a more pessimistic view of their illness. With respect to these constructs, the results are in line with the previous studies in this population, suggesting links between a higher sense of coherence and better illness perception being associated with a better quality of life [3, 13].

It is interesting to note that all variables related to PROs are also interrelated except for the physical activity subscale of the Health Behaviors Scale. It is possible that carrying out a cluster analysis on such a large sample has helped to uncover associations which were not explored in previous studies. It may also be that the presence of various demographic, clinical and psychosocial variables in the same person can have a cumulative effect which in turn results in global worsening of psychological functioning, which includes lower health perception, decreased quality of life and higher levels of anxiety and depression, and also an absence of those protective variables such a sense of coherence.

As noted previously, it is recommended that ACHD patients are provided with psychosocial screening accompanied by face to face clinical interviews [29, 30]. Resources to achieve this goal are currently not available in most ACHD centers in high-income countries. Therefore, identifying the higher risk patients may help inform the attending clinicians of their need for mental health care.

The challenges previously described for this specific ACHD population warrant specialised mental health care, especially considering that under-diagnosis and under-treatment of psychosocial concerns are present. Since resources in healthcare are limited and specialised psychosocial care is still not a standard practice for ACHD, being able to identify patients’ characteristics which are linked to psychosocial distress and a poor quality of life is of importance. More specifically, as suggested, it may be helpful to include psychologists during multidisciplinary medical meetings, organise specific and periodic psychosocial meetings in paediatric and adult cardiology and cardiac surgery units and if there are the available resources, screen all patients for psychosocial issues with the use of pertinent questionnaires and clinical interviews. [29, 31].

## Limitations

Although this is largest study of its kind, a few limitations must be acknowledged. First of all, causality cannot be determined because this is a cross-sectional study. Secondly, the patients included in the study are all being followed in CHD programs and therefore the results may not be generalizable to patients who are not being followed in CHD programs in participating countries. Thirdly, the patients who were not physically or mentally capable of completing the questionnaire are not captured by the study. Fourthly, it is not possible to determine the totality of possible factors that could impact on the PROs, such as undiagnosed syndromes or family history of mental problems. Finally, 260 patients could not be included in the analysis due to missing data.

## Conclusions

In this study it was reported that the majority of patients with CHD (i.e., clusters 1 and 2; 89.6%) have a good quality of life and are reasonably satisfied with their lives. They generally have a good health perception and psychological functioning when it comes to anxiety and depression. A minority of patients (i.e., 10.4%) fared less well on these constructs and had the following demographic and medical characteristics; higher percentage of females, more complex CHD, older age, lower level of education, more cardiac comorbidities (i.e., congestive heart failure, arrhythmias, and implanted cardiac devices, greater number of cardiac operations and catheterization), and more hospitalizations in the preceding year. Knowledge of these patient characteristics may help inform screening programs to identify and manage psychosocial difficulties in this population so as to provide timely interventions whenever possible.

## Ethical issues

The overarching study protocol was approved by the Institutional Review Board of the University Hospitals Leuven/KU Leuven (i.e., the coordinating center). Additionally, ethical approval was obtained by each participating center, if required. Although informed consent was obtained from all participants in most centers, there are some countries in which national legislation stipulates that written consent for survey studies is not required. Maintaining participant confidentiality is deemed a high priority. No personal health information (e.g., name, medical record number, or date of birth) is sent from the participating centers to the coordinating center. A unique patient study identification code consists of a two-digit center identification code followed by a three-digit patient identification number. For example, code 01–001 represents the first patient recruited from the first participating center. APPROACH-IS follows the recommendations of the Declaration of Helsinki. The authors of this manuscript have certified that they comply with the Principles of Ethical Publishing in the International Journal of Cardiology. The study protocol was recorded at ClinicalTrials.gov:NCT02150603.

## Data Availability

The current analyses are part of a large project, and that details on the project and publications coming from this project so far can be found using the following link: http://www.approach-is.net/theproject1.html
